# Living Atomically Dispersed Cu Ultrathin TiO_2_ Nanosheet CO_2_ Reduction Photocatalyst

**DOI:** 10.1002/advs.201900289

**Published:** 2019-05-24

**Authors:** Zaiyong Jiang, Wei Sun, Wenkang Miao, Zhimin Yuan, Guihua Yang, Fangong Kong, Tingjiang Yan, Jiachuan Chen, Baibiao Huang, Changhua An, Geoffrey A. Ozin

**Affiliations:** ^1^ State Key Laboratory of Biobased Material and Green Papermaking Qilu University of Technology Shandong Academy of Sciences Jinan 250353 P.R. China; ^2^ Department of Chemistry University of Toronto 80 St. George St. Toronto Ontario M5S 3H6 Canada; ^3^ State Key Laboratory of Silicon Materials and School of Materials Science and Engineering Zhejiang University Hangzhou Zhejiang 310027 P.R. China; ^4^ State key Laboratory of Crystal Materials Shandong University Jinan 250100 P.R. China; ^5^ Tianjin Key Laboratory of Organic Solar Cells and Photochemical Conversion School of Chemistry and Chemical Engineering Tianjin University of Technology Tianjin 300384 P.R. China

**Keywords:** atomically dispersed Cu, CO_2_ reduction, co‐catalysts, photocatalysis, TiO_2_ nanosheets

## Abstract

Supported atomically dispersed metals are proving to be efficacious photocatalysts for CO_2_ reduction to solar fuels. While being atom efficient, they suffer from being noble, rare, and costly (Pt, Pd, Au, Ag, Rh) and lacking in long‐term stability. Herein, all of these problems are solved with the discovery that atomically dispersed Cu supported on ultrathin TiO_2_ nanosheets can photocatalytically reduce an aqueous solution of CO_2_ to CO. The atomically dispersed Cu can be recycled in a straightforward procedure when they become oxidatively deactivated. This advance bodes well for the development of a solar fuels technology founded on abundant, low‐cost, nontoxic, atomically dispersed metal photocatalysts.

## 1 Introduction

Solar fuels from the reduction of CO_2_ require efficient photocatalysts.^1^, ^2^, ^3^, ^4^, ^5^, ^6^, ^7^, ^8^, ^9^, ^10^, ^11^, ^12^, ^13^, ^14^, ^15^, ^16^, ^17^, ^18^, ^19^, ^20^, ^21^, ^22^, ^23^, ^24^ To be commercially viable, their abundance, cost, and toxicity become an issue. An interesting way to circumvent this issue is through the use of single atom photocatalysts. The idea of exploiting the attributes of atom efficiency for photocatalytic CO_2_ reduction has been under intense scrutiny recently for supported single Pt, Pd, Au, Ag, and Rh atoms.^25^, ^26^, ^27^, ^28^, ^29^, ^30^, ^31^ While their photoactivity toward reducing CO_2_ is impressive, their rarity, cost, and long‐term stability could prove an impediment to commercialization. In this respect, Cu atoms would be ideal providing a synthetic route could be found and the challenges of long‐term chemical stability resolved.

Described herein, we present an innovative synthetic strategy for stabilizing atomically dispersed Cu on ultrathin TiO_2_ nanosheets and demonstrate that they not only can reduce CO_2_ to CO efficiently but can also be recycled in the process. This advance bodes well for the development of a solar fuels technology founded on abundant, low‐cost, nontoxic, atomically dispersed metal photocatalysts.

## 2 Results and Discussion

**Figure 1:**
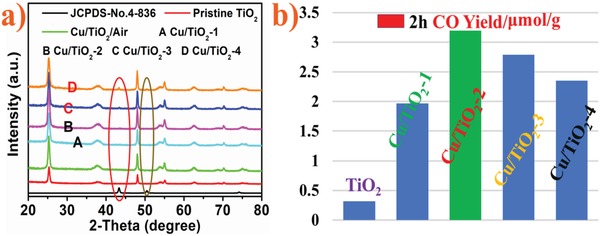
a) The PXRD patterns of samples Cu, TiO_2_, Cu/TiO_2_‐1,2,3,4 and b) CO yield of the samples after 2 h solar light irradiation.

Ultrathin TiO_2_ nanosheets were the semiconductor of choice for immobilizing atomically dispersed Cu. Attributes of the nanosheets include large surface area and fast transport of photogenerated charge carriers to the surface. It was discovered, however, that the in situ photodeposition method, commonly used for synthesizing noble metal cocatalysts (e.g., Pt, Ag, Pd, Au, Rh) failed to work on the nanosheets, seen from lack of color change, absence of power X‐ray diffraction (PXRD) and X‐ray photoelectron spectroscopy (XPS) diagnostics, and CO_2_ photocatalytic activity identical to the nanosheets alone (**Figure** **1**a and Figures S1–S3, Supporting Information). Fortunately, when the photodeposition was performed under CO_2_ reaction conditions, the colorless dispersion of TiO_2_ nanosheets changed to red and CO was formed as the only detected gas and liquid phase product (Figure S4, Supporting Information). This serendipitous observation suggested Cu atoms had deposited on the TiO_2_ nanosheets and exhibited CO_2_ reduction photoactivity.

Inspired by this result, we sought to optimize the production rate of CO by varying the loading of Cu atoms on the TiO_2_ nanosheets. Four samples were synthesized in this way by gradually increasing the concentration of precursor copper nitrate, denoted Cu/TiO_2_‐1, Cu/TiO_2_‐2, Cu/TiO_2_‐3, and Cu/TiO_2_‐4, corresponding with the amount of Cu(NO_3_)_3_ 3H_2_O (1, 3, 6, and 9 mg) respectively added to a 0.1 g TiO_2_ nanosheet aqueous suspension (100 mL). The photocatalytic CO_2_ reduction activities for all Cu/TiO_2_ samples were notably improved toward CO production compared to pristine TiO_2_. Among them, the Cu/TiO_2_‐2 displayed the highest activity of CO_2_ photoconversion after 2 h solar light irradiation, with a reaction rate about ten times (CO) higher than that of the pristine TiO_2_ (Figure 1b). Isotope ^13^CO_2_ tracing experiments analyzed by gas chromatography‐mass spectroscopy confirmed the photogenerated ^13^CO originated from the reduction of ^13^CO_2_ (Figure S5, Supporting Information).

PXRD patterns of samples Cu, TiO_2_, Cu/TiO_2_‐1,2,3,4 were recorded. The results show the presence of the anatase form of TiO_2_ (JCPDS no. 21–1272). At low Cu(NO_3_)_2_ 3H_2_O loadings, Cu, Cu_2_O, and CuO were absent. For high loading samples Cu/TiO_2_‐3 and Cu/TiO_2_‐4 weak diffraction peaks attributed to cubic metallic Cu (JCPDS no. 4–836) emerged (Figure 1a). Significantly, the low loading samples displayed the highest conversion rates of CO_2_ to CO. Collectively, these results suggest the state of Cu atom aggregation on the TiO_2_ nanosheets correlates with photocatalytic activity.

**Figure 2:**
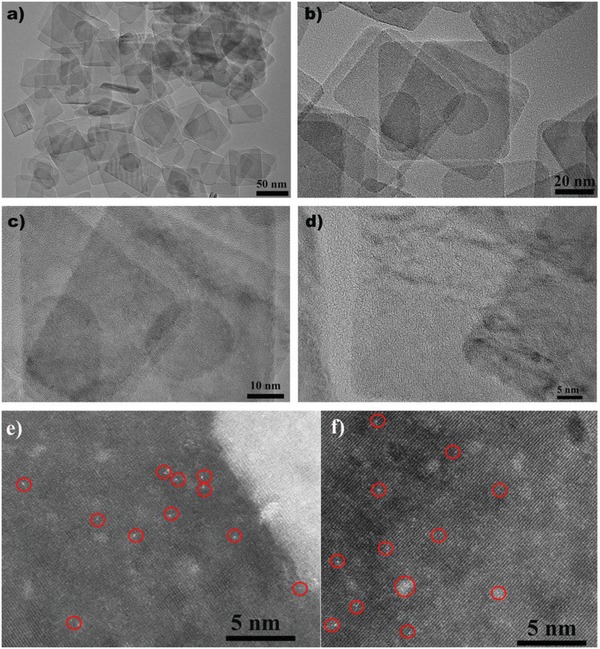
a–d) TEM images and e,f) atomic‐resolution STEM images of Cu/TiO_2_‐2. The atomically dispersed Cu and Cu clusters are highlighted with red circles.

Scanning electron microscopy images of TiO_2_ and Cu/TiO_2_‐1,2,3,4 samples show almost identical morphologies (Figures S6,S7, Supporting Information). Transmission electron microscopy (TEM) gives the impression the surface of the most photoactive Cu/TiO_2_ nanosheets is devoid of Cu atoms or clusters (**Figure** **2**a–d); however, spherical aberration‐corrected scanning transmission electron microscopy (STEM) shows this is not the case (Figure 2e,f). Images of atomically dispersed Cu are revealed under these high‐resolution imaging conditions as bright dots. Some Cu atom clusters on the surface of the TiO_2_ nanosheets can also be observed. A zoomed‐in image of a region abundant with such clusters shows that they are not crystalline but rather rich in Cu atoms in the vicinity of one another (Figure S8, Supporting Information). Energy‐dispersive X‐ray element mapping for Cu/TiO_2_‐2 provides additional evidenced for the Cu atoms and clusters with 0.79% coverage on the TiO_2_ nanosheets (Figures S9, S10, Supporting Information).

**Figure 3:**
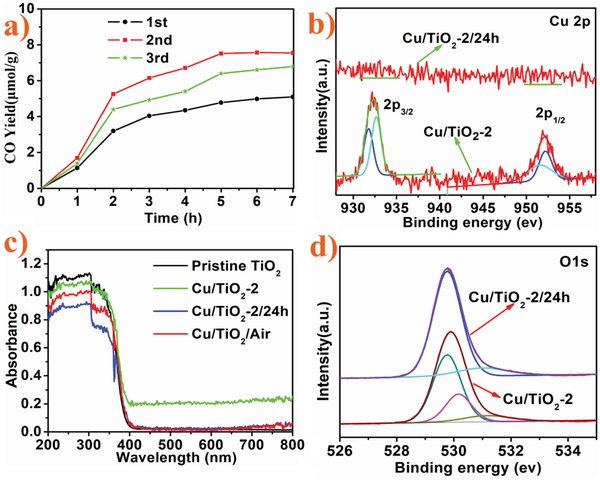
a) Photocatalytic CO_2_ → CO reduction activity of Cu/TiO_2_‐2 for three consecutive runs, b) high‐resolution XPS spectra of the Cu 2p region of Cu/TiO_2_‐2/24h and Cu/TiO_2_‐2, c) UV–vis absorption spectra of pristine TiO_2_, Cu/TiO_2_‐2, Cu/TiO_2_‐2/24 h, and Cu/TiO_2_/Air, d) high‐resolution XPS spectra of the O 1s region of Cu/TiO_2_‐2/24 h and Cu/TiO_2_‐2.

It was noted that after around 5 h on‐line photocatalytic testing, stability was becoming an issue (**Figure** **3**a), likely due to Cu atom oxidation. Curiously, opening the reactor to air for 24 h caused the dispersion to change its color from red to white (Figure S4b, Supporting Information). The white dispersion was filtered, washed with deionized water, dried at 60 °C for 6 h in a vacuum oven. The product, named Cu/TiO_2_‐2/24 h, was characterized by energy dispersive spectroscopy and XPS (Figure S11, Supporting Information and Figure 3b). No Cu signals were detected indicating loss of Cu from the surface of the TiO_2_ nanosheets, consistent with the unsuccessful attempt to photodeposit Cu onto TiO_2_ exposed to air mentioned earlier.

UV–vis absorption spectra of the pristine TiO_2_ nanosheets and Cu/TiO_2_‐2 were recorded (Figure 3c). Only Cu/TiO_2_‐2 exhibited some visible light absorption. Notably, the Cu/TiO_2_‐2/24 h sample had a similar absorption spectrum to pristine TiO_2_, consistent with the Cu having left the TiO_2_ nanosheets as mentioned above.

The aforementioned procedure suggests that the deactivated used form of Cu‐TiO_2_ can be restored to its original state and could therefore be recycled to its active state by simply resealing the photoreactor and continuing with the photocatalytic CO_2_ reduction again. Amazingly, the photocatalytic CO_2_ reduction activity was completely re‐instated, Figure 3a. Further six cycling tests each for 7 h showed no trend of deactivation (Figure S13, Supporting Information). This cyclic process could be repeated, giving the single Cu atom catalyst “living” status.

Experiments were designed to gain a deeper insight into the cyclic process for the most active Cu/TiO_2_ sample. High resolution O 1s XPS (Figure 3d), Cu 2p (Figure 3b), and Ti 2p (Figure S12, Supporting Information) provided some useful information. The O 1s XPS (Figure 3d) of Cu/TiO_2_‐2/24h displays two peaks located at 529.7 and 531.2 eV, which could be attributed to surface oxygen and hydroxyl groups of TiO_2_ nanosheets, respectively. A small peak at 530.2 eV might be from trace oxidation of Cu(0). The Cu 2p XPS appears to be composed of four Gaussian peaks, two at 951.5 and 931.8 eV, assigned to the Cu 2p_1/2_ and Cu 2p_3/2_ of Cu(0). Two peaks at 952.2 and 932.7 eV are likely associated with Cu(q+), (1 < q ≤ 2) species,^18^ illustrating the easy oxidation of highly dispersed Cu(0) on TiO_2_ nanosheets. This presence of Cu in Cu/TiO_2_‐2 is in clear contrast to Cu/TiO_2_‐2/24h in which Cu was detached from TiO_2_.

**Figure 4:**
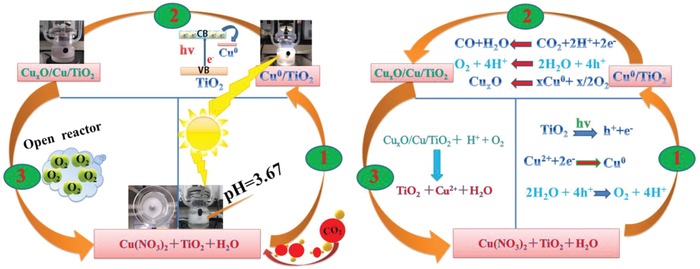
Schematic illustration of the cyclic process enabling an atomically dispersed Cu supported on ultrathin TiO_2_ nanosheet to function as a photocatalyst for the reduction of CO_2_ to CO.

With knowledge that the measured zeta potential of the TiO_2_ nanosheets is −2.12, a reaction pathway consistent with the above observations is proposed, **Figure** **4**. At pH 3.67 the copper nitrate precursor exists as Cu^2+^ and will be electrostatically adsorbed to the surface of the negatively charged TiO_2_ nanosheets. Under solar irradiation, photogenerated conduction band electrons of TiO_2_ will reduce surface Cu^2+^ to single atom Cu^0^. This is thermodynamically allowed based upon the redox potential for Cu^2+^/Cu = 0.34 eV versus standard hydrogen electrode, with conduction band of TiO_2_ around −0.29 eV. This process is only possible under a CO_2_ atmosphere to prevent oxidation of Cu^0^ → Cu^2+^ by O_2_ in H_2_O or air. Photogenerated electrons transported from the conduction band of TiO_2_ to Cu^0^ reduce the CO_2_ to CO and O^2−^, possibly via the HCO_3_^−^ and CO_2_^−^ intermediates previously observed by in situ diffuse reflectance infrared Fourier transform spectroscopy studies on Cu/TiO_2_ systems,^32^ while photogenerated holes in the valence band of TiO_2_ oxidize H_2_O to O_2_ and H^+^, the protons reacting with O^2−^ to form H_2_O and complete the photocatalytic cycle. The valence band XPS spectrum of Cu/TiO_2_‐2 was recorded from which the valence band maximum position (Figure S14, Supporting Information) is determined to be about 2.88 eV, the value further confirming the ability of producing oxygen. The Cu^0^ aids the separation of the electron hole charge carriers improving the photocatalytic activity in this process. Under photocatalytic reaction conditions, Cu^0^ becomes slowly oxidized and the activity decreases with time. Note that the oxidized Cu^q+^ (when q is <2) cannot be reduced by photoelectrons from TiO_2_ as its reduction potential is higher than the conduction band energy of TiO_2_. Yet the activity can be restored by opening the reactor to air then resealing it, following the cyclic process described above and illustrated in Figure 4.

## 3 Conclusion

In this work, it has been discovered that atomically dispersed Cu photodeposited on ultrathin TiO_2_ nanosheets can photocatalytically reduce an aqueous solution of CO_2_ to CO. The Cu atoms can be recycled in a straightforward procedure when they become oxidatively deactivated. This advance speaks well for the development of a solar fuels technology founded on abundant, low‐cost, nontoxic, atomically dispersed metal photocatalysts.

## 4 Experimental Section

*Materials*: Tetrabutyl titanate, Cu(NO_3_)_2_·3H_2_O, and 40% HF solution were purchased from the Sinopharm Chemical Reagent Corporation (Shanghai, China). All used experimental materials were without further purification, which were analytical grade in this study.

*Synthesis of TiO_2_ and Cu/TiO_2_ Composite Photocatalysts*: The TiO_2_ *nanosheets* were prepared according to the literature reported by Yu's group.^33^ The process of preparation was as follows: HF (3 mL) was added into 25 ml of tetrabutyl titanate and the mixed solution was continually stirred for 10 min. Subsequently, the resulting precursor suspension was kept in a 100 mL autoclave, which was maintained at 200 °C for 24 h. The autoclave was allowed to cool to room temperature, and then the as‐prepared sample was collected and washed via deionized water and absolute ethanol, respectively. The obtained product was dried at 60 °C for 6 h in an oven.

A series of Cu/TiO_2_ composite photocatalysts were prepared via an in situ online synchronous photodeposition method with different Cu(NO_3_)_2_·3H_2_O amount added 1, 3, 6, and 9 mg Cu(NO_3_)_2_·3H_2_O for 0.1 g TiO_2_ and 100 mL deionized water in CO_2_ atmosphere with the temperature controlled at 15 °C using cooling water circulation. The illumination time was 4 h and the corresponding products were noted as Cu/TiO_2_‐1/4 h, Cu/TiO_2_‐2/4 h, Cu/TiO_2_‐3/4 h, and Cu/TiO_2_‐4/4 h. After illumination treatment, the resulting suspension was filtered and washed using deionized water for three times. After that, the obtained Cu/TiO_2_ was dried at 60 °C in a vacuum oven.

*Characterization*: X‐ray powder diffraction was done on a Bruker AXS D8 advance powder diffractometer with Cu Kα X‐ray radiation. The particle sizes and morphologies of the samples were analyzed using a Hitachi S‐4800 microscope with an accelerating voltage of 7.0 kV. High‐resolution transmission electron microscopy measurements were performed using a JEOL‐2100 microscope. The UV–vis diffuse reflectance spectra were carried out on a Shimadzu UV 2550 recording spectrophotometer, which was equipped with an integrating sphere with BaSO_4_ as a reference. Fluorescence spectra were measured under 320 nm excitation. XPS was performed using a Thermo Fisher Scientific (ESCALAB 250) X‐ray photoelectron spectrometer and the result was charge corrected to the adventitious C 1s peak at 284.6 eV.

*Photocatalytic CO_2_ Reduction Evaluation*: The process of CO_2_ photoconversion is as follows: The sample (0.1 g) was dispersed into 100 mL water with vigorous stirring and continuously bubbled using high purity CO_2_ gas for 15 min. A 300 W Xe arc lamp (PLS‐SXE300, Beijing Trusttech Co., Ltd.) was applied as the light source and the temperature of reactor of CO_2_ photoconversion was kept at 15° via using cooling water circulation equipment. At the given time interval, the gas was collected and monitored via Varian CP‐3800 gas chromatograph (FID detector, Propark Q column). The pH of the system was not adjusted but measured as acidic, due to the dissociation of aqueous Cu(NO_3_)_2_·3H_2_O.

## Conflict of Interest

The authors declare no conflict of interest.

## Supporting information

Supplementary
