# Supplementary material for: Living Atomically Dispersed Cu Ultrathin TiO2 Nanosheet CO2 Reduction Photocatalyst
Source: Adv Sci (Weinh). 2019 May 24;6(15):1900289. doi: 10.1002/advs.201900289 (PMC6685599; doi:10.1002/advs.201900289)
Supplement: Supplementary file 1 — Supplementary [file ADVS-6-1900289-s001.pdf]

Copyright WILEY-VCH Verlag GmbH & Co. KGaA, 69469 Weinheim, Germany, 2019.

ADVANCED SCIENCE
Open Access

Supporting Information

for *Adv. Sci.,* DOI: 10.1002/advs.201900289

Living Atomically Dispersed Cu Ultrathin TiO_2 Nanosheet CO_2 Reduction Photocatalyst

Zaiyong Jiang, Wei Sun, Wenkang Miao, Zhimin Yuan, Guihua Yang, Fangong Kong, Tingjiang Yan, Jiachuan Chen,* Baibiao Huang, Changhua An, and Geoffrey A. Ozin**

Supporting Information

Living Atomically Dispersed Cu Ultrathin TiO_2 Nanosheet CO_2 Reduction Photocatalyst

Zaiyong Jiang,,# Wei Sun,# Wenkang Miao, Zhimin Yuan, Guihua Yang, Fangong Kong, Tingjiang Yan, Jiachuan Chen,* Baibiao Huang, Changhua An, Geoffrey A. Ozin**

[#] These authors contribute equally to this work.

Dr. Z. Jiang, W. Miao, Dr. Z. Yuan, Prof. G. Yang, Prof. F. Kong, Prof. J. Chen,
State Key Laboratory of Biobased Material and Green Papermaking, Qilu University of Technology, Shandong Academy of Sciences, Jinan 250353, P.R. China.
E-mail address: zaiyongjiang@qlu.edu.cn, chenjc@qlu.edu.cn

Dr. Z. Jiang, Dr. W. Sun, Prof. T. Yan, Prof. G. A. Ozin,
Department of Chemistry, University of Toronto, 80 St. George St., Toronto, Ontario, M5S 3H6, Canada.
E-mail: gozin@chem.utoronto.ca

Dr. W. Sun
State Key Laboratory of Silicon Materials and School of Materials Science and Engineering, Zhejiang University, Hangzhou, Zhejiang 310027, PR China

Prof. B. Huang
State key Laboratory of Crystal Materials, Shandong University, Jinan 250100, PR China.

Prof. C. An
Tianjin Key Laboratory of Organic Solar Cells and Photochemical Conversion, School of Chemistry and Chemical Engineering, Tianjin University of Technology, Tianjin 300384, PR China.

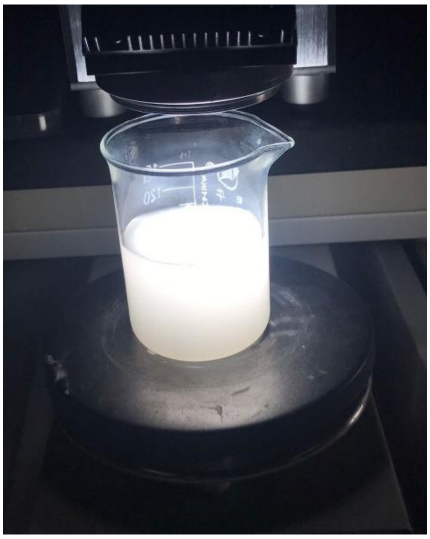

Figure S1. A photo of the experiment of in-situ photodeposition in air. The color of suspension remained white.

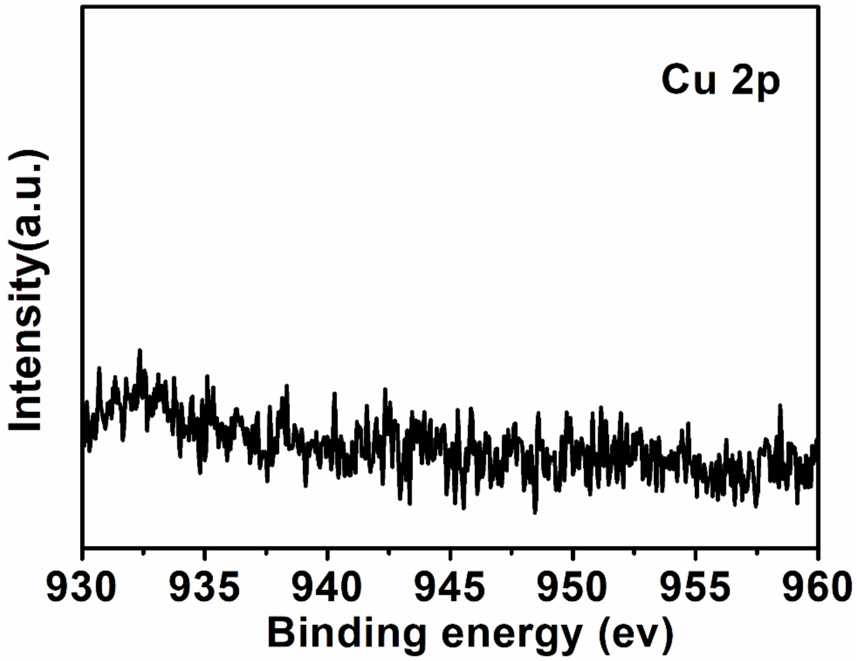

Figure S2. High-resolution XPS spectra of the Cu 2p region of Cu/TiO_2/Air.

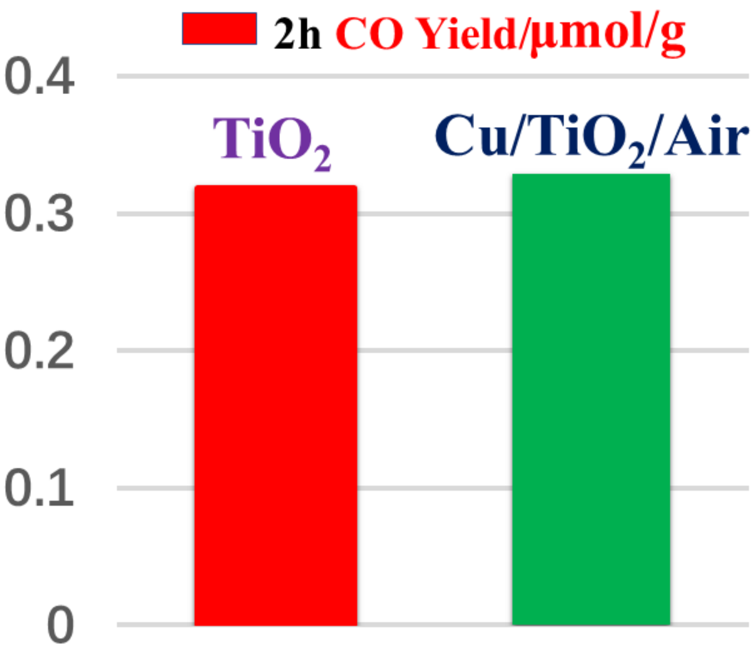

Figure S3. Photocatalytic CO_2 reduction activities of pristine TiO_2 and Cu/TiO_2/Air.

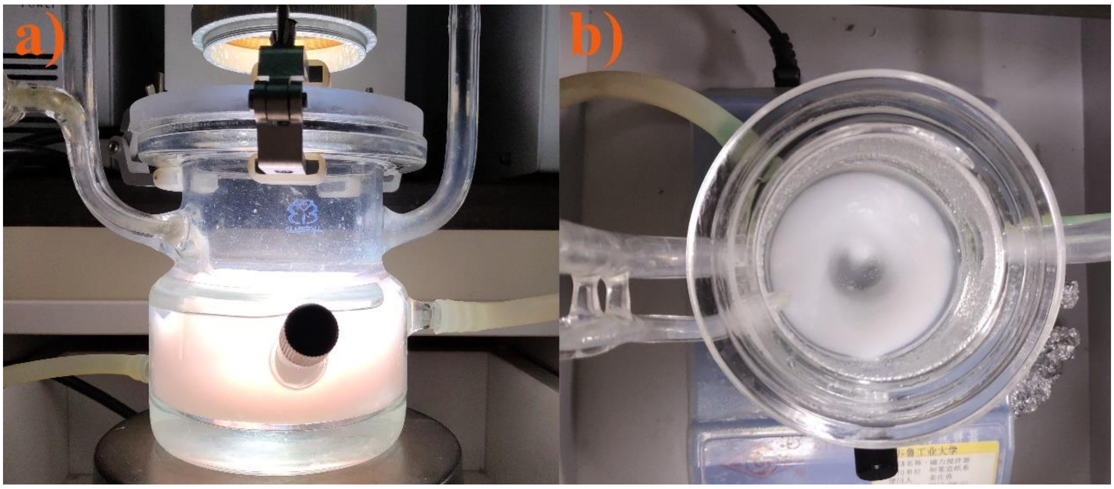

Figure S4. Photos taken (a) after completing the experiment of in-situ photodeposition in CO_2 atmosphere, showing the red-pink colour of the suspension solution and (b) after the suspension was stirred in air showing the mere white colour.

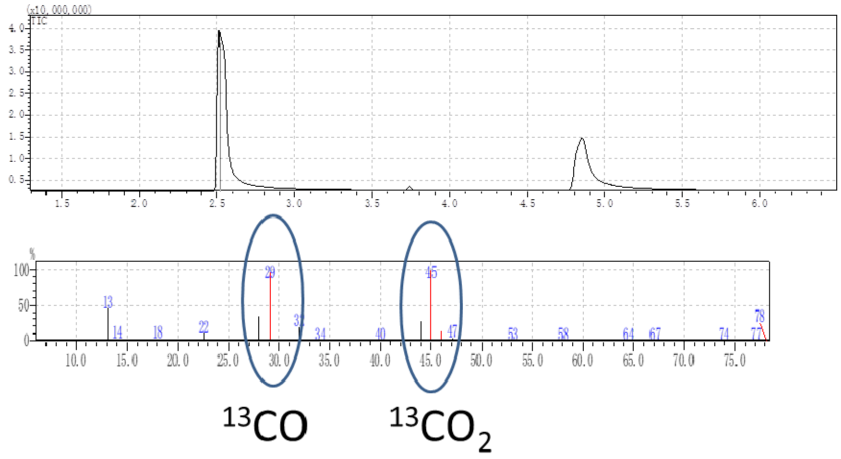

Figure S5. Isotope (^{13}C) tracing experiments.

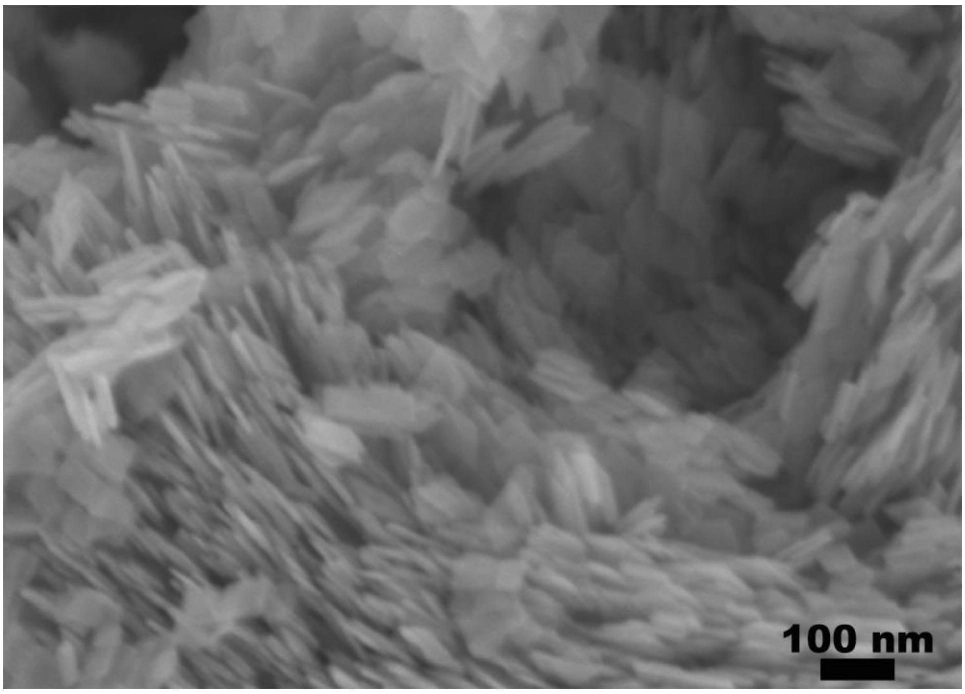

Figure S6. An SEM image of pristine TiO_2 nanosheets.

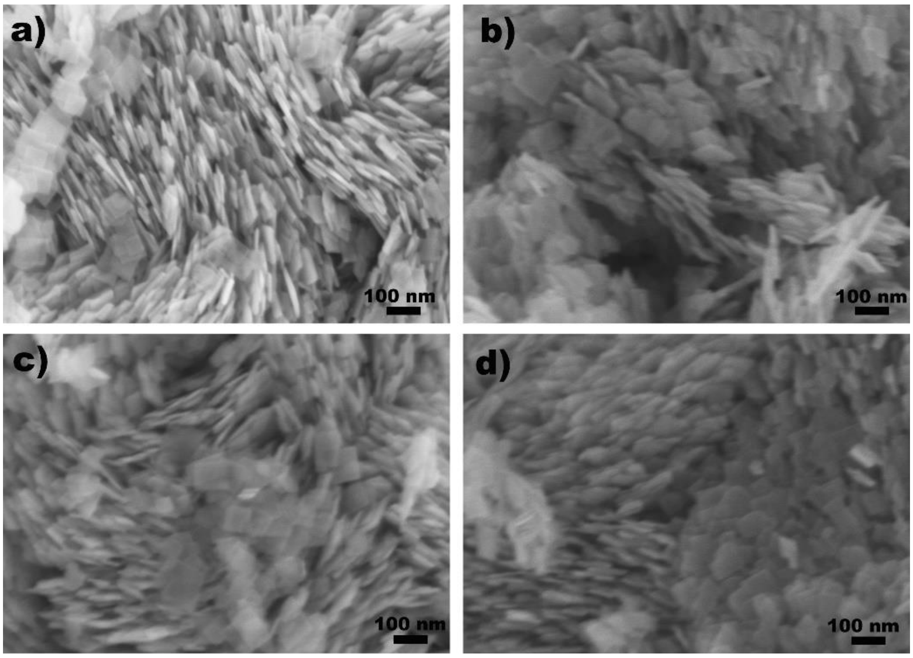

Figure S7. SEM images of (a) Cu/TiO_2-1, (b) Cu/TiO_2-2, (c) Cu/TiO_2-3 and (d) Cu/TiO_2-4.

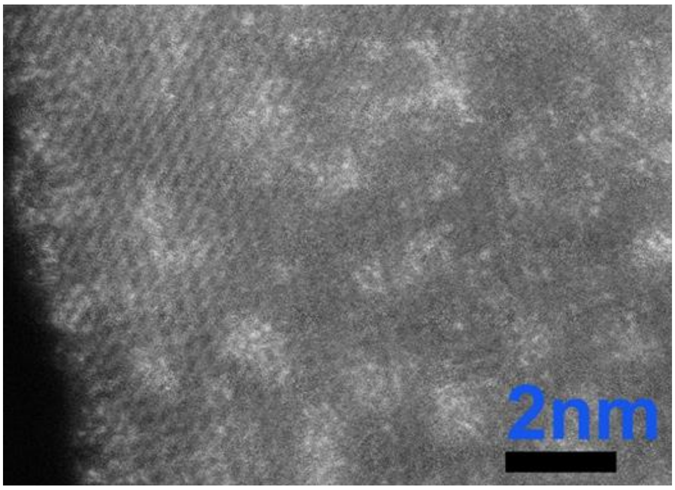

Figure S8. A zoomed-in atomic-resolution STEM image of a Cu-rich region of Cu/TiO_2-2, showing the clusters are not crystalline.

Element	Weight %	Atomic %
C K	5.79	11.44
O K	42.50	63.01
TiK	51.18	25.35
CuK	0.53	0.20

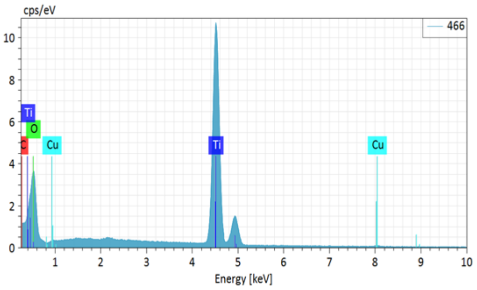

Figure S9. EDS nanlysis of Cu/TiO_2-2.

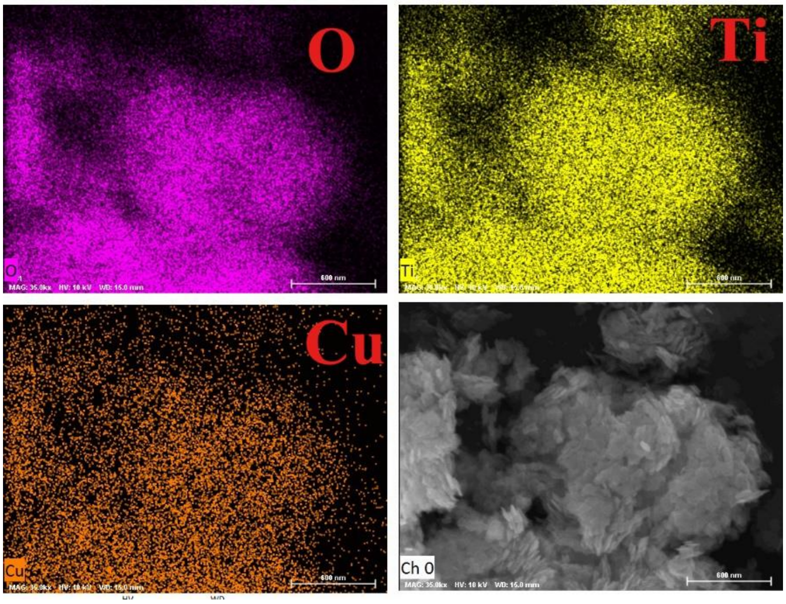

Figure S10. EDS mapping images of Cu/TiO_2-2.

Element	Weight %	Atomic %
C K	13.77	24.93
O K	39.69	53.94
TiK	46.54	21.13

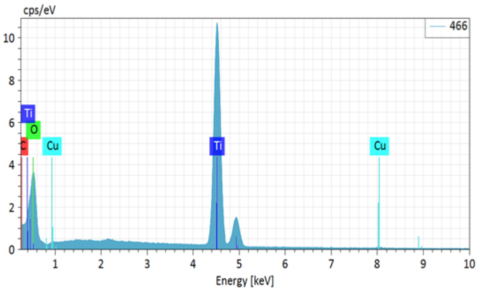

Figure S11. EDS analysis of Cu/TiO_2-2/24h.

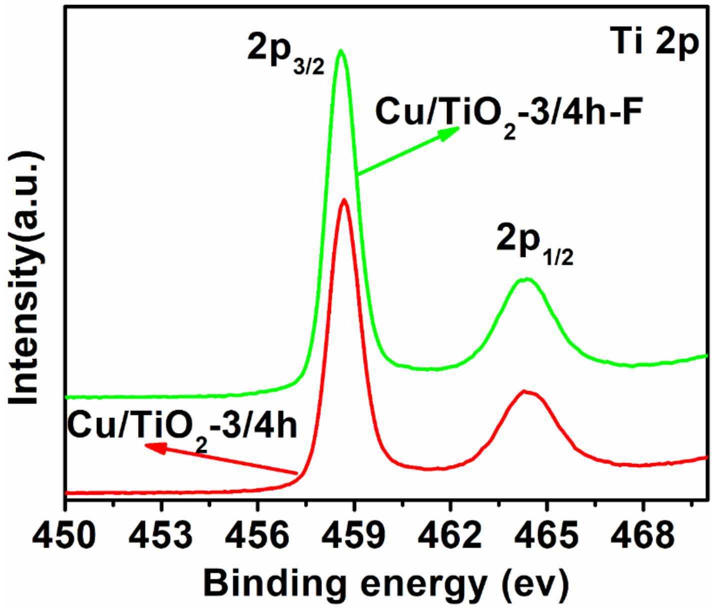

Figure S12. High-resolution XPS spectra of Ti 2P region, showing the similar binding energies of Ti^{4+} are 458.6 and 464.4 eV, respectively, in both samples.

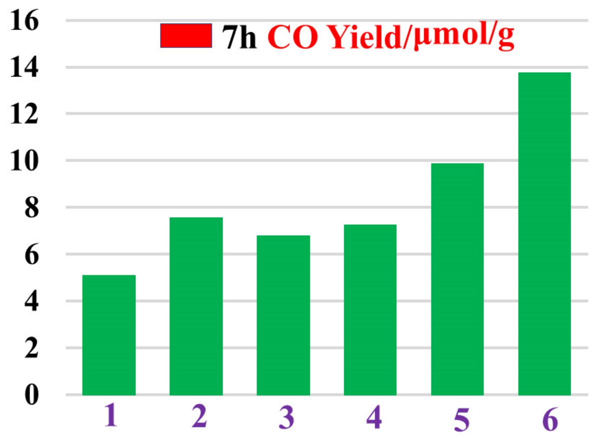

Figure S13. Photocatalytic CO_2 → CO reduction activity of Cu/TiO_2-2 for six consecutive runs.

In each cycle, the reaction solution was continuously bubbled with high purity CO_2 gas for 15 min. The CO production was not deactivated but rather activated over cycling, likely due to the enrichment of dissolved CO_2.

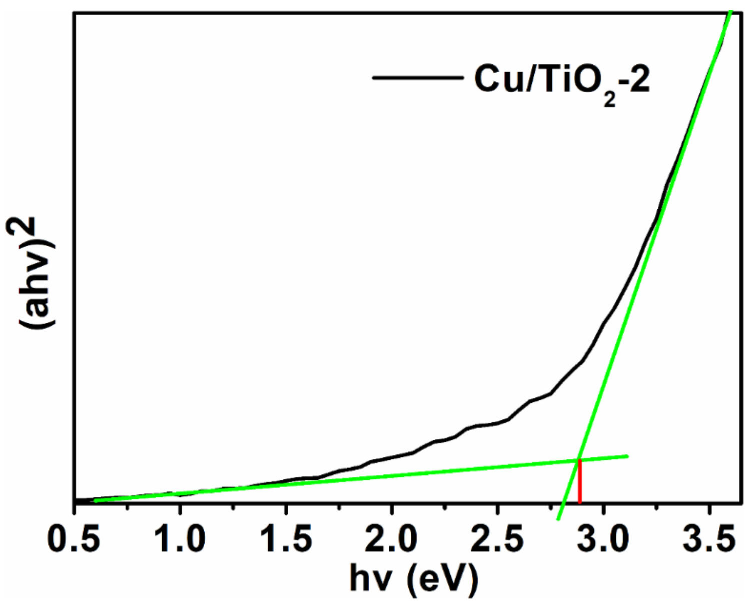

Figure S14. The valence band XPS spectrum of Cu/TiO_2-2.

.
